# Uridylation and adenylation of RNAs

**DOI:** 10.1007/s11427-015-4954-9

**Published:** 2015-10-27

**Authors:** JianBo Song, Jun Song, BeiXin Mo, XueMei Chen

**Affiliations:** 1 Shenzhen Key Laboratory of Microbial Genetic Engineering, College of Life Science, Shenzhen University, Shenzhen 518060, China; 2 Key Laboratory of Optoelectronic Devices and Systems of Ministry of Education and Guangdong Province, College of Optoelectronic Engineering, Shenzhen University, Shenzhen 518060, China; 3 Department of Biochemistry and Molecular Biology, College of Science, Jiangxi Agricultural University, Nanchang 330045, China; 4 Department of Botany and Plant Sciences, Institute of Integrative Genome Biology, University of California, Riverside CA 92521, USA; 5 Howard Hughes Medical Institute, University of California, Riverside, CA 92521, USA

**Keywords:** Uridylation, adenylation, miRNA, pre-miRNA, U6 snRNA, histone mRNA, rRNA

## Abstract

The posttranscriptional addition of nontemplated nucleotides to the 3′ ends of RNA molecules can have a significant impact on their stability and biological function. It has been recently discovered that nontemplated addition of uridine or adenosine to the 3′ ends of RNAs occurs in different organisms ranging from algae to humans, and on different kinds of RNAs, such as histone mRNAs, mRNA fragments, U6 snRNA, mature small RNAs and their precursors etc. These modifications may lead to different outcomes, such as increasing RNA decay, promoting or inhibiting RNA processing, or changing RNA activity. Growing pieces of evidence have revealed that such modifications can be RNA sequence-specific and subjected to temporal or spatial regulation in development. RNA tailing and its outcomes have been associated with human diseases such as cancer. Here, we review recent developments in RNA uridylation and adenylation and discuss the future prospects in this research area.

Nontemplated addition of nucleotides to RNA 3′ ends has long been known, with the addition of CCA to the 3′ ends of tRNAs and mRNA polyadenylation being the earliest described examples [1,2]. Relatively more recently, studies reveal that nontemplated 3′ nucleotide addition to eukaryotic RNAs is a more widespread phenomenon. Conserved posttranscriptional processes result in 3′ uridylation or adenylation of histone mRNAs, mRNA fragments, tRNAs, rRNAs, U6 snRNA, and mature small RNAs and their precursors, and these modifications are often associated with functional outcomes [3,4]. Here we discuss the evidence that reveals the functions of RNA 3′ uridylation and adenylation, and explore instances where the role of these modifications is currently less clear.

## 1 3′ uridylation affects RNA synthesis, degradation and function

### 1.1 Uridylation of histone mRNAs

Histone mRNAs are the only known metazoan mRNAs that are not polyadenylated, ending instead in a conserved stem-loop sequence. The stem-loop-binding protein (SLBP) participates in nearly all aspects of histone mRNA metabolism, such as pre-mRNA processing [5], mRNA export [6], translation [7,8], and degradation [9]. Histone mRNAs are rapidly degraded at the end of the S phase of the cell cycle or when DNA replication is inhibited [10,11].

**Figure 1:**
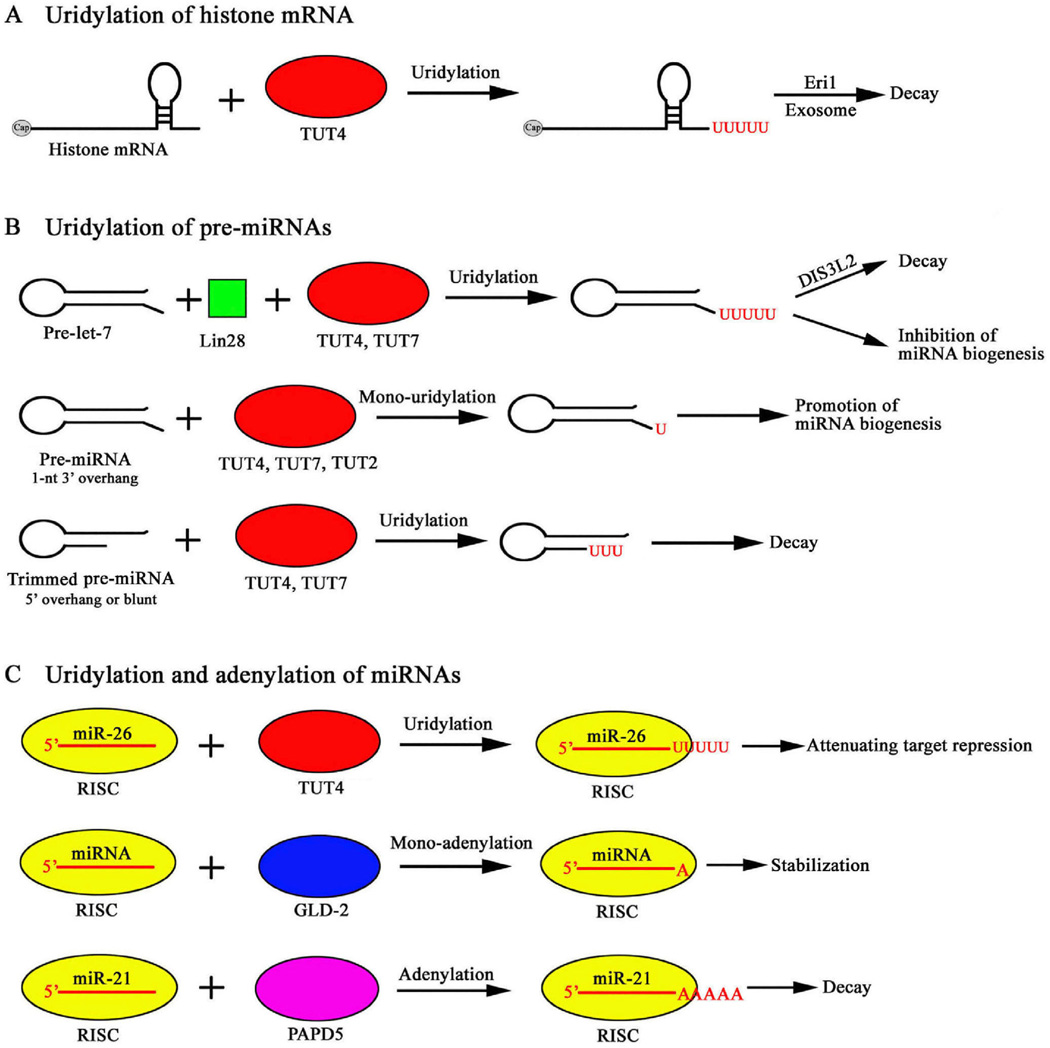
A summary of the substrates and outcomes of RNA uridylation and adenylation in humans.

In human cells, histone mRNA degradation begins with the assembly of a complex of factors, including SLBP and Exoribonuclease 1 (Eri1), on the 3′ end of the mRNA, resulting in the addition of uridine to the 3′ end of the histone mRNA. Following the oligouridylation event, the heteroheptameric Lsm1–7 complex binds to the oligo(U) tail to trigger subsequent histone mRNA degradation via both 5′–3′ and 3′–5′ RNA decay pathways [10,12]. The C-terminal extension of Lsm4 interacts directly with the 3′ end of the histone mRNP and this interaction is required for efficient histone mRNA degradation [13]. ZCCHC11 is the terminal uridylyl transferase responsible for human histone mRNA uridylation following inhibition or completion of DNA replication [14] (Table 1 and Figure 1). Eri1, as an exonuclease, acts on oligouridylated histone mRNAs and trims histone mRNA 3′ ends into the stem-loop [15]. Recently, deep sequencing revealed that histone mRNAs are degraded 3′–5′ in two phases: degradation into the stem loop by Eri1 followed by degradation by the exosome-associated 3′–5′ exonuclease PM/Scl-100 [16]. If the nuclease stalls during either phase of the degradation process, further degradation is primed by re-uridylation. Multiple oligouridylation events may be required for 3′–5′ degradation of histone mRNAs on polyribosomes [16].

### 1.2 Uridylation of microRNA (miRNA)-directed 5′ cleavage products

By regulating gene expression in a sequence-specific manner, miRNAs play important roles in numerous biological processes. miRNAs repress target gene expression through translational inhibition as well as RNA degradation in both plants and animals, but the mechanisms of miRNA-triggered RNA degradation are largely different in plants and animals [17,18]. In animals, miRNAs trigger deadenylation followed by decapping and exonucleolytic degradation of target mRNAs [19]. Nearly all plant miRNAs and very few animal or animal viral miRNAs guide the precise endonucleolytic cleavage of target transcripts [20–22]. The 3′ cleavage fragments are degraded in the of 5′–3′ direction by the exonuclease XRN4 in *Arabidopsis* [23], but degradation of the cleaved 5′ mRNA fragments is more complex and entails 3′ uridylation [24].

**Figure 2:**
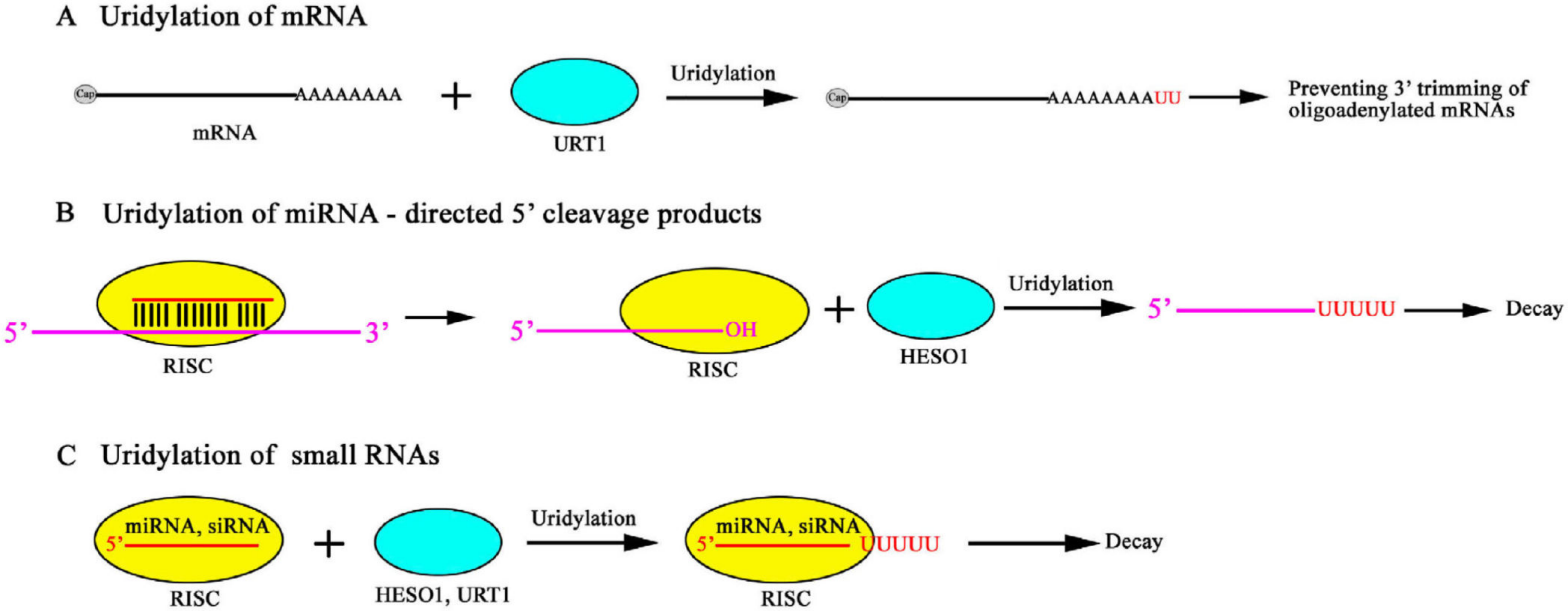
A summary of the substrates and outcomes of RNA uridylation in *Arabidopsis*.

The presence of an oligo(U) signature posttranscriptionally added to miRNA-directed 5′ cleavage products in species as diverse as *Arabidopsis*, mouse, and Epstein-Barr virus implies that uridylation has general importance [24]. In mammalian cell extracts, uridylation of the 3′ end of an RNA promotes their decapping relative to an RNA lacking the uridine tract [25]. In addition to promoting decapping, the nontemplated oligo(U) tail prevents 3′–5′ exonucleolytic decay to ensure 5′–3′ directional degradation [25]. In *Arabidopsis*, HESO1 uridylates the 5′ fragments resulting from miRNA-guided cleavage of target RNAs to trigger their degradation, and AGO1, the effector protein of miRNAs, is associated with HESO1 *in vivo* [26] (Table 1 and Figure 2B).

### 1.3 Uridylation of snRNAs

The U6 small nuclear RNA (snRNA) is a member of the evolutionarily conserved snRNA class within the eukaryotic spliceosome. Mammalian U6 snRNA is heterogeneous in size due to nontemplated 3′ uridylation [27]. A major form contains five terminal U residues and a 2′, 3′ cyclic phosphate; minor forms contain up to 12 U residues and a 3′ OH [27,28]. These forms probably represent the dynamic nature of the U6 3′ end in the spliceosome, as these forms are all present in the U4/U5/U6 tri-snRNPs [28], and are the result of two opposing enzymatic activities that elongate and trim the 3′ end. U6-TUTase is a terminal uridylyl transferase that posttranscriptionally 3′ oligouridylates U6 snRNA [29,30], whereas USB1 is a distributive 3′–5′ exoribonuclease that posttranscriptionally removes uridine and adenosine nucleotides from the 3′ end of U6 snRNA [31]. As the length of the U tail as well as the presence or absence of the 2′, 3′ cyclic phosphate modulates the affinity of U6 to RNA binding proteins such as La and the heteroheptameric Lsm2–8 complex [32], both of which associate with U6 during snRNP maturation and recycling, the uridylation of U6 RNA is considered an integral process in U6 RNA metabolism and splicing.

### 1.4 Uridylation of small RNAs

3′ uridylation of mature small RNAs was first found in plants. *Arabidopsis* miRNAs or small interfering RNAs (siRNAs) are methylated on the 2′ OH of the 3′ terminal ribose by the methyltransferase HUA ENHANCER 1 (HEN1) [33,34]. In *hen1* mutants, both miRNAs and siRNAs undergo 3′ truncation and 3′ uridylation, leading to their decline in abundance [35]. Later, 3′ uridylation was found to also occur to siRNAs and/or PIWI-interacting RNAs (piRNAs) in *hen1* mutant animals, such as *Tetrahymena* [36]. *C. elegans* [37–39], *Drosophila* [40,41], zebra fish [42] and mouse [43]. Small RNA 3′ uridylation is not restricted to *hen1* mutants; it occurs in wild type cells at a lower frequency. Studies using high throughput sequencing to profile small RNAs revealed 3′ nontemplated nucleotide addition, mainly uridylation and adenylation, to mature miRNAs from viruses [44], *Chlamydomonas reinhardtii* [45], *Drosophila* [46], mouse [47], and human cells [48].

In recent years, an increasing number of terminal nucleotidyl transferases (TUTases) that uridylate small RNAs have been identified. In humans, Zcchc11 (TUT4) uridylates the cytokine-targeting miRNA miR-26b; the uridylation appears to attenuate the target repression activity of this miRNA such that Zcchc11 promotes the expression of cytokine genes [49] (Table 1 and Figure 1). Zcchc6 (TUT7) and Zcchc11 (TUT4) uridylate a small set of miRNAs with a common sequence motif [48] (Table 1 and Figure 1). Depletion of these TUTases in cultured human cells leads to a reduction in 3′ monouridylation, and interestingly, a concomitant increase in nontemplated 3′ monoadenylation of these miRNAs, without affecting their abundance [48,50]. In *C. elegans*, the nucleotidyl transferase CDE-1 uridylates siRNAs bound by the argonaute protein CSR-1 to prevent their over accumulation and loading into other argonaute proteins; CDE-1 is essential for proper meiotic and mitotic chromosome segregation [51] (Table 1). MUT68, a nucleotidyl transferase in the alga *Chlamydomonas reinhardtii* contributes to the presence of nontemplated uridine residues at the 3′ ends of small RNAs and loss of function in *MUT68* results in elevated miRNA and siRNA levels [45] (Table 1). *Arabidopsis* HEN1 SUPPRESSOR1 (HESO1) is a nucleotidyl transferase responsible for most of the small RNA uridylation in *hen1* mutants. HESO1 prefers UTP as the substrate nucleotide, and is completely inhibited by 2′-*O*-methylation in the substrate RNA [52,53]. Loss of function in *HESO1* leads to increased miRNA accumulation in *hen1* mutants [52,53]. UTP: RNA uridylyltransferase (URT1) is a functional paralog of HESO1 that is responsible for the remainder of small RNA uridylation in *hen1 heso1* mutants [54]. URT1 and HESO1 have distinct substrate preferences *in vitro* and act cooperatively to tail different forms of the same miRNAs *in vivo* [54,55] (Table 1 and Figure 2).

A surprising finding is that during the regulation of a target, the small RNA itself may be subjected to regulation by the target, which results in the posttranscriptional addition of a nontemplated uridine to the miRNA [56]. This indicates that small RNA regulatory pathways may have built-in feedback regulation. In *Drosophila*, the introduction of artificial RNAs with a high degree of sequence complementarity to miRNAs leads to the 3′ trimming and 3′ tailing of the cognate miRNAs [57].

### 1.5 Uridylation of pre-miRNAs

The precursors to the let-7 miRNA were first found to undergo uridylation [58]. Later, high throughput sequencing revealed that pre-miRNA 3′ uridylation is not limited to pre-let-7 and occurs in a developmentally regulated manner [58–62]. Studies with pre-let-7 show that the outcomes of pre-miRNA uridylation are two fold: triggering pre-miRNA degradation or promoting their processing into miRNAs (discussed below).

In human embryonic stem cells, TUT4, a nucleotidyl transferase, acts in concert with the RNA-binding protein Lin28 to uridylate pre-let-7 [58,62]. After the nuclear export of pre-let-7, Lin28 recognizes a sequence motif in the RNA loop and recruits TUT4 to add an oligo(U) tail of 10–30 nt to the 3′ terminus of pre-let-7. The tail renders pre-let-7 resistant to Dicer processing and may facilitate its decay [58,62] (Table 1 and Figure 1). The related nucleotidyl transferase TUT7 acts redundantly with TUT4 in this process–simultaneous knockdown of TUT7 and TUT4 leads to increased let-7 levels in embryonic stem cells [63]. The E3 ligase Trim25 binds to the conserved terminal loop of pre-let-7 and acts as an RNA-specific cofactor to activate TUT4 for more efficient Lin28-mediated uridylation [64]. Degradation of oligouridylated pre-let-7 requires the 3′–5′ exonuclease Dis3L2, which prefers U-ending RNAs as substrates [65–68].

However, in differentiated cells, pre-let-7 uridylation has a different outcome. In mouse P19 teratocarcinoma cells, which stop expressing Lin28 upon *in vitro* differentiation, profiling of pre-miRNAs revealed Lin28-dependent pre-let-7 oligouridylation and Lin28-independent pre-let-7 monouridylation [59]. In human somatic cells, Lin28 is not expressed and the nucleotidyl transferases TUT7, TUT4, and TUT2 monouridylate pre-let-7; this converts pre-let-7 with a 1 nt 3′ overhang to a better Dicer substrate with a 2 nt 3′ overhang and thus enhances dicer processing [69] (Table 1 and Figure 1).

Related to the function of triggering degradation, pre-miRNA uridylation also plays a role in pre-miRNA quality control. High throughput sequencing of pre-miRNAs revealed oligouridylation of 3′ resected pre-let-7, suggesting that degradation intermediates of pre-let-7 need to be uridylated for further degradation [59]. In TUT4/TUT7-depleted cells, argonaute-bound pre-miR-106b and pre-miR-18a had a higher fraction of species with blunt or 5′ overhangs, which are likely to be degradation intermediates [60] (Table 1 and Figure 1). This suggests that uridylation helps to turnover argonaute-bound, non-productive pre-miRNAs. High throughput sequencing revealed that mirtron pre-miRNAs, which are generated from intron splicing rather than Drosha processing, are preferentially uridylated as compared to canonical pre-miRNAs [70,71]. Two recent studies identified the *Drosophila* nucleotidyl transferase Tailor as the enzyme that uridylates mirtron pre-miRNAs [72,73]. The specificity of Tailor for mirtron pre-miRNAs could be explained by the preference for a 3′ G in the substrate RNA by this enzyme, as introns released from splicing should end with a 3′G [72,73] (Table 1).

## 2 3′ Adenylation affects RNA synthesis and degradation

### 2.1 Adenylation of miRNAs

Adenylation of miRNAs was first discovered in *hen1* mutants in *Arabidopsis* [35], but the impact of miRNA adenylation was unclear. In animals, uridylation and adenylation are the two most frequent miRNA 3′ modifications as revealed by high throughput sequencing [46,74]. In vertebrates, many small RNAs can be maternally deposited by the mother or expressed in the zygote to regulate early embryonic development. Profiling of small RNAs during early development in zebra fish revealed widespread miRNA 3′ uridylation and 3′ adenylation, and such modifications were found to undergo developmental stage-specific regulation [75]. Profiling of small RNAs in cells and exosomes (secreted vesicles from cells) revealed the enrichment of 3′ adenylation in cells and 3′ uridylation in exosomes [76].

Adenylation of miR-122 has a stabilizing effect on this miRNA. GLD-2 is a cytoplasmic, non-canonical poly(A) polymerase responsible for the 3′ terminal monoadenylation of miR-122 and other miRNAs in mouse livers and human fibroblast cells [77,78]. In GLD2 knockout mice, miR-122 levels were selectively reduced, suggesting that 3′ adenylation stabilizes the miRNA [77]. In human fibroblasts, miR-122 is also monoadenylated and stabilized by GLD2 [78], and the hepatitis B virus may inhibit the process of miR-122 adenylation [79]. Adenylation of miRNAs may have a widespread functional impact in humans, because miRNAs in a variety of human cells are modified by adenylation [77,78,80,81].

But the stabilizing effect of 3′ adenylation on miR-122 may not be extrapolated to other miRNAs. Another study examined the effects of GLD-2 knockdown in human THP-1 cells (GLD-2 was referred to as PAPD4 in this study) and showed that GLD-2 is responsible for the 3′ adenylation of many miRNAs [46] (Table 1 and Figure 1). However, the reduction in 3′ adenylation did not correlate with increased miRNA levels; instead it correlated with reduced expression of miRNA target genes, suggesting that adenylation reduced miRNA activity [46].

A study on human miR-21, an oncogenic miRNA implicated in numerous human diseases, concluded that adenylation of miR-21 leads to its destabilization [82]. In human THP-1 cells, 3′ adenylation of miR-21 is caused by the non-canonical poly(A) polymerase PAPD5 rather than GLD-2. Knocking down either PAPD5 or the exonuclease PARN led to increased miR-21 levels and reduced miR-21 species with 3′ trimming, suggesting that PAPD5-mediated adenylation of miR-21 triggers 3′–5′ digestion of the miRNA by PARN [82] (Table 1 and Figure 1).

### 2.2 Adenylation of mRNA

In eukaryotic cells, the cotranscriptional addition of a poly(A) tail to the 3′ ends of mRNA molecules is nearly universal; the poly(A) tail protects mRNAs from degradation and facilitates their translation from yeast to higher eukaryotes [1]. But in recent years, it was found that in some cases, polyadenylation leads to the degradation of mRNAs. For example, in human cells, the β-globin pre-mRNA is cotranscriptionally cleaved, oligoadenylated, and degraded by the 3′–5′ nuclease exosome [83]. The mouse serum albumin (MSA) gene also undergoes cotranscriptional cleavage of the pre-mRNA near the 3′ end of the gene, and some of the transcripts are also oligoadenylated and degraded by the exosome [83]. Pre-mRNA degradation may represent a secondary role for RNA adenylation in mammals. In *Chlamydomonas*, MUT68 oligoadenylates 5′ RNA fragments generated by small RNA-mediated cleavage and leads to their degradation by a 3′–5′ exonuclease, most likely the exosome [84].

### 2.3 Adenylation of rRNAs

Although rRNAs are not produced by RNA polymerase II and thus are not subjected to cotranscriptional polyadenylation as do mRNAs, in yeast, a small fraction of precursor rRNAs is posttranscriptionally modified at their 3′ ends by the addition of a poly(A) tail *in vivo* [85,86]. As the levels of polyadenylated precursor rRNAs dramatically increase when Rrp6p, a component of the nuclear exosome, is mutated, rRNA polyadenylation is thought to trigger exosome-mediated degradation as a surveillance mechanism to remove improperly processed rRNAs [85]. The 5′-exoribonuclease Rat1p and its associated protein Rai1p are also responsible for the degradation of poly(A)^+^ pre-rRNAs [86]. 5-fluorouracil (5FU), a chemotherapeutic compound for the treatment of solid tumors, was found to inhibit this exosome-dependent surveillance pathway that degrades polyadenylated precursor rRNAs [87].

## 3 Specificity and regulation of RNA uridylation and adenylation

As discussed above, uridylation or adenylation of RNAs is a widespread phenomenon found for different RNA species and in many organisms, but these posttranscriptional processes exhibit specificity and undergo regulation. For example, miRNA 3′ uridylation and adenylation exhibit sequence specificity—some miRNAs are predominantly adenylated while others are predominantly uridylated [79,88]. This is consistent with the fact that some nucleotidyl transferases (such as *Arabidopsis* URT1 and HESO1 and *Drosophila* Tailor) exhibit a preference for the 3′ nucleotide in its substrate RNA [55,72,73] (Table 1 and Figure 2). RNA-binding proteins also contribute to the recruitment of nucleotidyl transferases to specific substrates as discussed above [62,64]. miRNA 3′ tailing appears to undergo developmental regulation. For example, during early development in *Drosophila*, the levels of uridylated miRNAs are higher, but in mature tissues, the levels of adenylated miRNAs are higher [89]. Patterns of uridylation and/or adenylation of miRNAs in healthy tissues are different from cancerous tissues [79].

## 4 Conclusions and perspective

**Figure 3:**
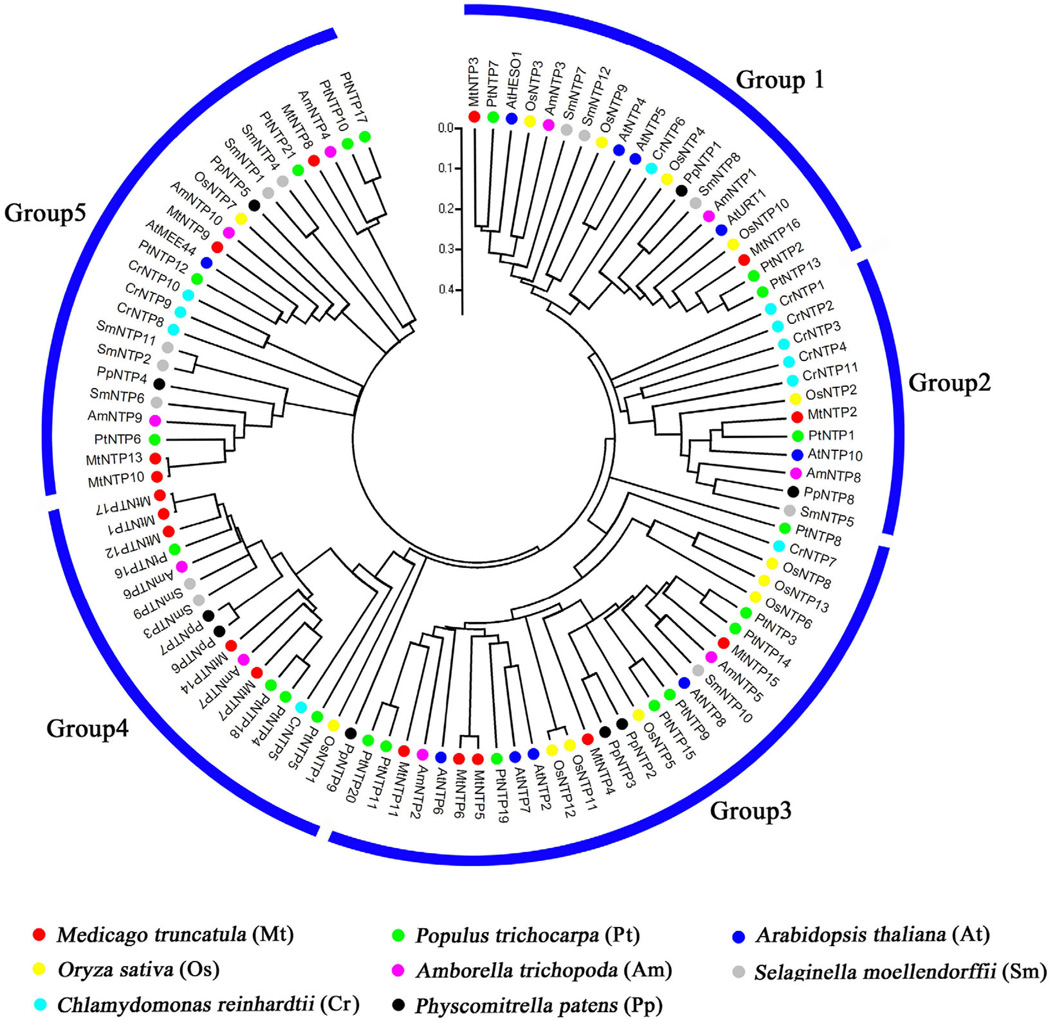
A phylogenetic tree of the nucleotidyl transferase protein (NTP) family from *Arabid*opsis *thaliana* (At), *Oryza sativa* (Os), *Amborella trichopoda* (Am), *Medicago truncatula* (Mt), *Populus trichocarpa* (Pt), *Selaginella moellendorffii* (Sm), *Physcomitrella patens* (Pp) and *Chlamydomonas reinhardtii* (Cr). The potential NTPs from various organisms were retrieved by searches using the PFam nucleotidyl transferase domain (PF01909) [91] as the query against the protein databases for these organisms at Phytozome (http://phytozome.jgi.doe.gov). The searches were performed with the HMMER3 pipeline [92,93]. The full-length NTP protein sequences were aligned by CLUSTAL X 2.0 [94], and the alignments were used to generate an unrooted phylogenetic tree with MEGA 5.1 [95], using the p-distance method and a bootstrap value of 1,000. Evolutionary distance is indicated by the scale bar inside the figure. The NTPs used in the analysis are listed in Supplemental Table S1.

Uridylation and/or adenylation are universal and conserved RNA modifications that have major impacts on the degradation, synthesis and mode of action of RNAs. Given the diverse types of modifications, such as mono- and oligo-uridylation and mono- and oligo-adenylation, and the diverse RNA substrates that undergo the modifications, it is hard to generalize on the functional outcomes of the modifications. A common theme is perhaps that a stretch of homo-oligomeric nucleotides, either A or U, tends to lead to RNA degradation by allowing exonucleases to overcome RNA secondary structures or protection by RNA binding proteins (ribosome, argonaute, etc.). Monouridylation or monoadenylation may impart different outcomes on different RNAs. Key to RNA uridylation or adenylation are nucleotidyl transferases, whose preference for UTP or ATP dictates the nature of the tail to be added and whose processivity may determine whether one or a number of nucleotides are added. Plant and animal genomes encode multiple nucleotidyl transferases [55,62] (Figure 3), many of which have not been characterized. For example, there are 10 putative nucleotidyl transferases in *Arabidopsis* (Figure 3), among which only HESO1 and URT1 have been studied [52–55,90] (Table 1 and Figure 2). Studying the enzymatic properties and biological functions of nucleotidyl transferases will lead to a better understanding of 3′ tailing in RNA metabolism.

## Figures and Tables

**Table 1:** Nucleotidyl transferases with known uridylation and/or adenylation activity from various species

Name	Alternative name(s)	Organism	Substrates*	Activity*	Potential effects*
HESO1	At2g39740	*Arabidopsis*	miRNAs, siRNAs, miRNA-directed 5′ cleavage products	Uridylation	Decay
URT1	At2g45620	*Arabidopsis*	miRNAs, siRNAs mRNAs	Uridylation Uridylation	Decay Stabilization
CDE-1	CID-1, PUP-1, KD10D2.3, Ce5	*C. elegans*	siRNAs	Uridylation	Decay
MUT68	polβNTase, Chlredraft_149294	*Chlamydomona* *reinhardtii*	miRNAs, siRNAs	Uridylation	Decay
Tailor		*Drosophila*	Mirtron pre-miRNAs	Uridylation	Inhibition of biogenesis
GLD-2	PAPD4, Hs1	Human	miRNAs	Mono-adenylation	Stabilization
ZCCHC11	TUT4, PAPD3, Hs3	Human	Histone mRNAs, pre-let-7, trimmed pre-miRNAs miR-26 Certain pre-miRNAs	Uridylation Uridylation Mono-uridylation	Decay Attenuating target repression Maturation
PAPD5	TUT3, TRF4-2	Human	miR-21	Adenylation	Decay
TUT2		Human	Certain pre-miRNAs	Mono-uridylation	Maturation
ZCCHC6	TUT7, PAPD6, Hs2	Human	Pre-let-7, trimmed pre-miRNAs Certain pre-miRNAs	Uridylation Mono-uridylation	Decay Maturation

* , “Activity” and “Potential effects” refer to the “Substrates” in the same row.
